# Influence of inherent minerals on metalworking fluids sludge pyrolysis: Products characterization and heavy metals behavior

**DOI:** 10.1016/j.heliyon.2024.e26256

**Published:** 2024-02-10

**Authors:** Guidan Zhu, Xingdong Wang, Xuan Yin, Mengmeng Zhu, Jiaying Li, Ling Cao, Zhiyang Sun, Hehua Zeng

**Affiliations:** a School of Chemistry and Chemical Engineering, Changji University, Changji, 831100, China; b Department of Civil Engineering, 23 College Walk, Monash University, Victoria, 3800, Australia; c College of Mechanical and Electrical Engineering, Beijing University of Chemical Technology, Beijing, 100029, China; d Xinjiang Qinghua Energy Group Co., Ltd., Yining, 835100, China

**Keywords:** Metalworking fluids sludge, Pyrolysis, Product, Characterization, Heavy metals

## Abstract

Safely and appropriately disposing of metalworking fluids sludge (MFS) remains a challenge owing to its highly hazardous properties, this work investigated MFS pyrolysis at various temperatures (500, 600, 700, 800, and 900 °C) for energy recovery and safety treatment of MFS. The experimental results indicated that inherent minerals at higher temperatures could enhance the gas yields and promote the qualities of oil and gas from MFS pyrolysis. The highest pyrolysis gas yield was achieved at 18.86 wt% after MFS pyrolysis at 900 °C. GC-MS analysis revealed that the inherent minerals facilitated a decrease in oxygenated and nitrogenated compounds within the oil, while simultaneously leading to a substantial increase in hydrocarbon contents. Notably, the highest content of aromatics (61.16%) was attained during pyrolysis at 900 °C. Moreover, inherent minerals improved carbon sequestration and the characteristics of biochar during the MFS pyrolysis. The leaching contents of heavy metals in biochars were reduced, thereby reducing the heavy metals associated environmental risk. This research suggests that the pyrolysis process was a promising approach for simultaneous energy recovery and MFS disposal with low environmental risk.

## Highlights

• Pyrolysis technology was used for MFS disposal and energy recovery.
• Synergistic effects of inherent minerals in MFS on products composition were conducted.
• Inherent minerals in MFS promoted the qualities of oil and gas.
• Enhanced biochar properties can be achieved after MFS pyrolysis.
• The directly toxic heavy metals in biochar were lower after MFS pyrolysis.

## Graphical abstract

**Figure:**
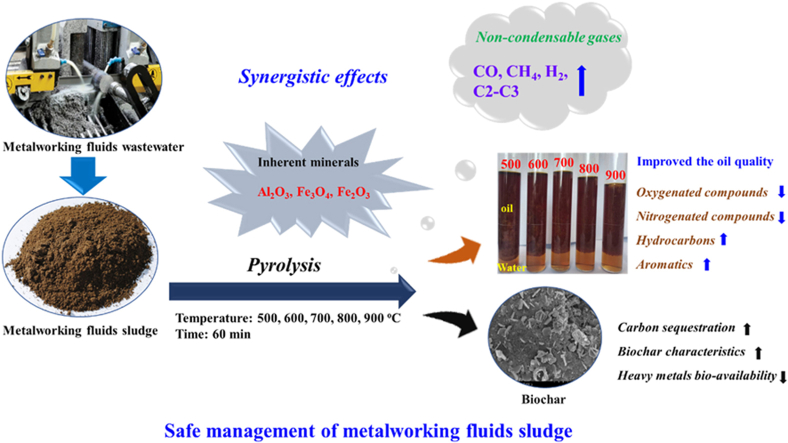

## 1 Introduction

Metalworking fluids sludge (MFS) is typically classified as hazardous solid waste, which is produced from metalworking processes involving cutting fluids [1,2]. The MFS composition is very complex, containing a mixture of hazardous substances, including heavy metals, toxic organic compounds, dioxins, biocides and additives, and other contaminants [3]. Currently, the primary methods for disposing of MFS include landfills and incineration. Nevertheless, landfills can pose a significant risk of groundwater contamination due to leachate-containing contaminants from MFS, and land resources are also growing progressively limited [4]. On the other hand, incineration is a well-established technology for the safe disposal of MFS, but it does face challenges related to pollutant emissions and high operational costs [5]. Therefore, it is imperative to employ a harmless and low-carbon method for safely treating MFS.

Pyrolysis is recognized as a low-carbon technology for hazardous waste treatment, and it is increasingly drawing attention due to its capability to reduce greenhouse gas emissions, sequester carbon, immobilize heavy metals, and facilitate energy and resource recovery, from the perspectives of low-carbon practices and environmental protection [6]. The pyrolysis of sludge also can convert organic matter to bio-fuels (oil and gas) and high-quality biochar with a porous structure [7]. The sludge-based biochar is a carbonaceous material with significant carbon content and abundant adsorption sites [6,8]. Hence, it is frequently employed as a soil amendment to mitigate CH_4_ and N_2_O emissions, enhance water retention, and neutralize soil acidity [[9], [10], [11]]. The composition and chemical forms of heavy metals accumulated in sludge-based biochar are essential factors that influence its properties and suitability for various environmental applications. Several studies have investigated the distribution and chemical form evolution of heavy metals during sludge pyrolysis [12,13]. Heavy metals in sludge have the potential to be redistributed into biochar, bio-oil, and syngas during pyrolysis, and this redistribution is mainly governed by pyrolysis conditions and the types of heavy metals involved [14,15]. Previous studies have indicated that certain metals (Zn, As, Pb, and Cd) readily undergo volatilization into the oil and gas when the pyrolysis temperature exceeds 600 °C [16,17]. Conversely, more than 90% of other metals (Cu, Cr, and Ni) exhibit a propensity to concentrate in the biochar following the pyrolysis of sludge [18]. Significantly, the effective immobilization of a greater quantity of heavy metals in biochar during sludge pyrolysis is exclusively achieved under elevated temperatures, leading to the transformation of heavy metals in sludge from bioavailable forms to more stable states [16].

The impact of inherent minerals on sludge pyrolysis characteristics has garnered significant attention. Researches have affirmed that these inherent minerals can act as catalysts in chemical reactions, affecting pyrolysis performance, the pyrolytic products properties, and the immobilization capacities of heavy metals in biochar [[19], [20], [21]]. For instance, Liu et al. [22] found that the Fe–Mg layer double hydroxides can suppress the emission of N-/S-containing pollutants in pyrolysis products. Santos et al. [23] found that CaO, F_2_O_3_, MgO, and Al_2_O_3_ in steel slag effectively promoted the quality and the properties of oil and gas during waste biomass pyrolysis. The inherent minerals presence in sludge also can improve the physicochemical characteristics of biochar and accelerate the conversion of initial hydrocarbons into higher-value products [24]. Mineral elements in sludge also can chemically react with heavy metals during the sludge pyrolysis process, resulting in the formation of more stable crystalline compounds [[25], [26], [27], [28]]. This transformation process enhances the stability of heavy metals by converting them from their bioavailable states into more stable forms. Introducing alkaline catalysts such as CaO, Fe_2_O_3_, and NaOH into the sludge at a moderate pyrolysis temperature can also promote the immobilization of heavy metals [29,30]. This immobilization process was intricately associated with the porous structure and co-crystalline compounds present in biochar derived from sludge.

In comparison to other sludges, the MFS contains higher levels of mineral components, such as oxides of Al, Fe, and Si [31]. We hypothesized that the MFS pyrolysis process has a good potential to promote the qualities of pyrolysis products and immobilize heavy metals by using its intrinsic mineral oxides. However, up to now, the detailed roles of inherent minerals on pyrolytic product properties and heavy metals behavior during MFS pyrolysis still remains unclear. Therefore, in this work, the pyrolysis of MFS at various temperatures (500, 600, 700, 800, and 900 °C) was conducted in the tube furnace. The main objectives of this study were to (1) investigate the influences of pyrolysis temperatures and inherent minerals on the composition of the pyrolytic product; (2) reveal the synergistic effects of inherent minerals on the biochar characteristics; and (3) evaluate the total concentrations and leaching properties of heavy metals in biochar. This is the first study to research the effect of inherent minerals on the MFS pyrolysis performance, which would provide new insight into the influences of inherent minerals on MFS pyrolysis characteristics, product composition, and heavy metals immobilization.

## 2 Materials and methods

### 2.1 Materials

The MFS was collected from a waste metalworking fluids wastewater treatment plant in Urumqi City, China. The collected MFS was initially sorted to remove large metal fragments and other particles (>1 mm), and then oven-dried at 105 °C for 24 h to eliminate moisture, finally grounded and sieved to fine particles of size≤0.15 mm and placed in a desiccator before the pyrolysis procedure. All chemicals used in this study, including hydroxylamine hydrochloride, hydrogen peroxide, ammonium acetate, and potassium bromide, were of analytical grade (AR). The acids employed for sample digestion, including nitric acid, perchloric acid, hydrofluoric acid, and acetic acid, were of guaranteed grade (GR) to ensure there was no secondary pollution of heavy metals. All solutions were prepared using deionized water. High-purity nitrogen (99.999%) was utilized as the atmosphere gas during the pyrolysis procedure.

### 2.2 Experimental procedure

**Fig. 1:**
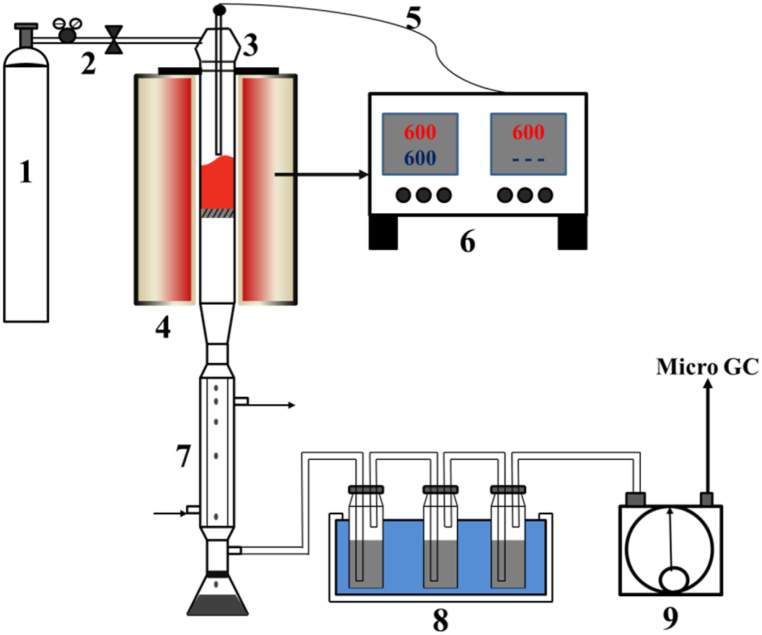
Scheme of the pyrolysis apparatus (1, high-pressure N_2_; 2, flow control; 3, horizontal quartz tube; 4, heated furnace; 5, thermocouple; 6, temperature controller; 7, condenser; 8, acetone trap; 9, gas collector).

The pyrolysis of MFS was carried out using a custom-built fixed-bed pyrolysis furnace, as depicted in Fig. 1. MFS pyrolysis at various temperatures (500, 600, 700, 800, and 900 °C) was performed following the methods detailed in our previous publication [32]. During each pyrolysis process, 50 g of dried MFS was initially placed into a quartz reaction tube, and then subjected to a 60-min purging with 99.999% pure N_2_ to remove any oxygen before pyrolysis. Throughout the entire pyrolysis process, a constant flow of 100 ml/min of N_2_ was maintained. The pyrolysis temperature was increased from room temperature to the desired temperature at a rate of 20 °C/min and was then held steady for 60 min. The condenser section was adjusted to −15 °C to facilitate the condensation of pyrolysis oil and water. Three acetone washing bottles were placed in an ice bath (˂ 0 °C) to act as secondary oil absorbers. The resulting biochars derived from MFS pyrolysis at various temperatures (500, 600, 700, 800, and 900 °C) were denoted as MC500, MC600, MC700, MC800, and MC900, respectively. The collected condenser was employed for the static separation of oil and water. And the acetone-washed oil was subjected to rotary evaporation at 18 °C to completely eliminate the acetone. The resulting two sections of oil constitute the overall quality of oil utilized in the pyrolysis process. The non-condensable gases were ultimately gathered in a gas bag for further analysis of their composition.

### 2.3 Product characteristics and analysis

The composition of non-condensable gases, including CO_2_, CO, CH_4_, H_2_, and C_2_–C_3_, was examined using gas chromatography (GC, GC-2014C, Shimadzu, Japan). The oil compositions obtained in this work were analyzed using gas chromatography-mass spectrometry (GC–MS, Agilent 7890A/5975C, USA). The compositions of CHNS elements and mineral elements in raw MFS and its biochars were detected using the elemental analyzer (Vario EL cube, Hanau, Germany) and the X-ray fluorescence spectrometry (ZSX Primus III+, Rigaku, Japan). The pH values of raw MFS and its biochars were determined using a digital pH meter (UB-10, Denver, USA). The ash contents of the samples were assessed following the standard method GB/T 12496.3–1999 (MEP, 1999). The surface areas and porosity of samples were tested by the N_2_ adsorption-desorption isotherms using a surface area analyzer (ASAP 2460, Micromeritics, USA). The crystal structure of the minerals in samples was assessed using the X-ray diffractometer (SmartLab SE, Rigaku, Japan) within an angular range from 5^o^ to 85^o^. The FTIR (Nicolet iS10, Thermo Fisher, USA) was utilized to characterize the surface functional group properties of samples. The morphological features of the samples were examined with a high-resolution SEM (Regulus 8230, Hitachi, Japan).

### 2.4 Heavy metals analysis

The MFS and its biochars were firstly digested using a mixed acid solution (HNO_3_: HClO_4_: HF = 4: 4: 3, v/v) with the assistance of a microwave digestion system (Mars 6, CEM, USA). The Toxicity Characteristic Leaching Procedure (TCLP) is a widely employed method for examining the immobilization of heavy metals and predicting their potential leachability [26]. Leachates from the MFS and its biochars were extracted using an acetic acid solution (pH: 2.88) with a liquid/solid ratio of 20:1. The extraction process involved shaking for 18 h at 25 °C in a shaking incubator. The resulting filtrates underwent digestion with H_2_O_2_/HNO_3_ to eliminate dissolved organics. All solutions obtained from both the TCLP extraction and microwave digestion were filtered through a 0.22 μm nylon filter and adjusted to a consistent volume of 100 mL using a 2.0% HNO_3_ solution before the test. The heavy metal concentrations in all solutions were analyzed using inductively coupled plasma mass spectrometry (ICP-MS) (7500CX, Agilent Technologies, USA).

## 3 Results and discussions

### 3.1 Pyrolysis products analysis

#### 3.1.1 Pyrolysis products yield

**Fig. 2:**
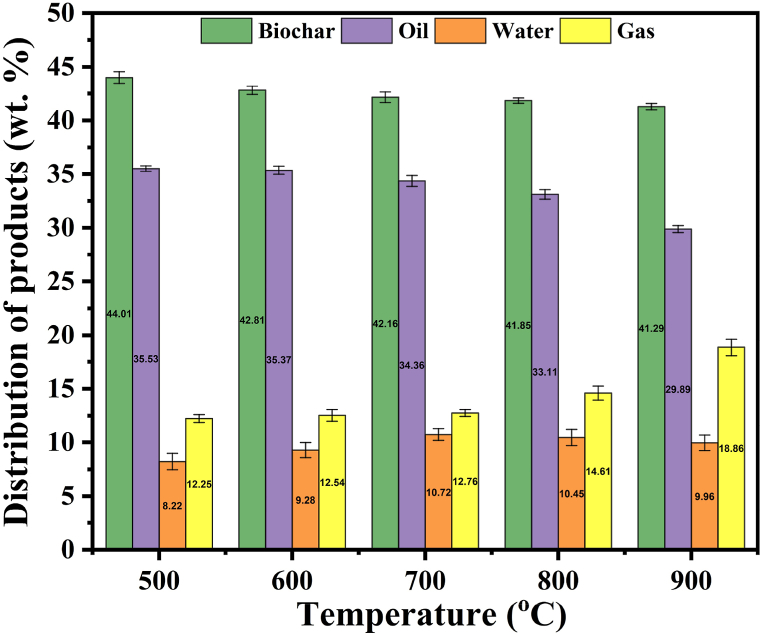
Product yields under different pyrolysis temperatures.

The yields of pyrolysis products including biochar, oil, water, and non-condensable gas were shown in Fig. 2. The yields of biochar and oil slowly decreased from 44.01 wt% and 35.53 wt% to 41.29 wt% and 29.89 wt%, respectively, as the temperature increased from 500 to 900 °C. Similar results were also observed in another research conducted by Zuo et al. [33], where the biochar yield exhibited a sharp decrease from 300 to 500 °C and a slight decrease from 500 to 700 °C during MFS pyrolysis. This phenomenon may be attributed to that the decomposition of organic substances in the MFS mainly occurs before 500 °C during the pyrolysis process. While the yield of gas increased with rising temperature and reached a maximum of 18.86 wt% at 900 °C. Elevated temperatures and the presence of inherent minerals in MFS facilitated the decomposition of organic components, consequently promoting the production of non-condensable gas [24]. The water yields initially increased with temperature (≤700 °C) and reached a maximum of 10.72 wt% at 700 °C, which could be attributed to the inherent minerals favoring the cleavage and reforming of carboxyl and carbonyl groups [34], thereby enhancing water production. The water yields slightly dropped to 9.96 wt% as the temperature increased to 900 °C.

#### 3.1.2 Pyrolysis oil composition analysis

**Fig. 3:**
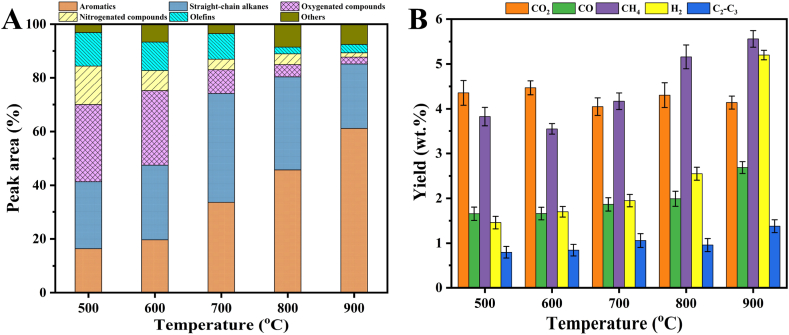
Influence of temperature on GC-MS components distribution of oil (A) and gas composition yields (B).

Fig. 3A displayed the main components in the oil, as identified in the GC-MS chromatograms. These components can be categorized into oxygenated compounds, straight-chain alkanes, aromatics, nitrogenated compounds, olefins, and others. As indicated in Fig. 3A, the pyrolysis temperature had a notable impact on the composition of oil during MFS pyrolysis. The oil derived from a low temperature of 500 °C contained high percentages of oxygenated compounds (28.67%) and nitrogenated compounds (14.36%). As the pyrolysis temperature increased, the content of oxygenated and nitrogenated compounds decreased rapidly to very low levels of 2.55% and 1.59% in the oil derived from MFS pyrolysis at 900 °C, while the hydrocarbons (including straight-chain alkanes, aromatics, and olefins) significantly increased from 53.87% at 500 °C to 88.21% at 900 °C. Notably, a substantial increase in aromatics with a maximum value of 61.16% was observed in the oil resulting from MFS pyrolysis at 900 °C. Similar results were also discovered in a study on oily sludge pyrolysis [35], which indicated that the addition of mineral catalysts promoted the cracking reactions of complex oxygenated and nitrogenated macromolecular compounds, resulting in an increased production of aromatic hydrocarbons. These findings suggest that the inherent minerals in MFS can significantly improve the quality of the oil.

#### 3.1.3 Pyrolysis gas composition analysis

Fig. 3B displayed the gas composition yields at various temperatures, with the major gas components being CO_2_, CO, CH_4_, H_2_, and C_2_–C_3_. Except for CO_2_, which remained relatively constant in the range of 4.05–4.47 wt%, the yield of other gas components generally increased with rising temperature. This trend can be ascribed to the enhanced occurrence of cracking, dehydrogenation, and decarbonylation reactions at high temperatures. The MFS pyrolysis at 900 °C achieved the highest yields of CO (2.69 wt%), CH_4_ (5.56 wt%), H_2_ (5.20 wt%), and C_2_–C_3_ (1.38 wt%). This increase may be attributed to the influence of inherent minerals at high temperatures, which could intensify cracking reactions, including the cracking of alkyl chains and methoxy groups in organic components within MFS. These findings imply that the MFS pyrolysis process can recover energy by producing gases with high levels of energy-containing compounds, such as H_2_, CO, and hydrocarbons.

### 3.2 Properties of the biochar

#### 3.2.1 General properties

The general properties of the MFS and its biochars derived from various temperatures including pH value, ash content, and elemental composition were listed in Table 1. Compared to other sludges, MFS had a notably low pH of 4.71. After pyrolysis conversion to biochar, the pH value significantly increased and showed a positive correlation with the pyrolysis temperature, which can be attributed to the decomposition of acidic surface functional groups and the accumulation of mineral oxides [16]. A higher pH value can notably enhance the capacity for immobilizing heavy metals in biochar. The ash content in biochar also increased as the pyrolysis temperature increased, mainly owing to the retention of the majority of mineral constituents from MFS following pyrolysis (Table 1). The pyrolysis of MFS resulted in a decline of C, H, N, S, and O in the biochars due to the thermal decomposition of organic matter, as demonstrated in Table 1. As the pyrolysis temperature increased, the levels of H, N, and O in the biochar decreased, while the S content remained relatively stable. Conversely, the C content in the biochar exhibited an increase with higher pyrolysis temperatures, suggesting an enhancement in carbon sequestration attributed to the presence of alkali metals in minerals from MFS [36]. The H/C molar ratio is commonly used as an index for assessing aromaticity and the O/C molar ratio is employed to evaluate the energy density in biochar. The H/C and O/C molar ratios of biochars decreased as the temperature increased during MFS pyrolysis, suggesting an improvement in the aromatic structure and energy density of the resulting biochars.

**Table 1:** The general properties of MFS and its biochars.

Item	pH	Ash (%)	C (%)	H (%)	N (%)	S (%)	O (%)	H/C	O/C	S_BET_ (m^2^/g)
MFS	4.71	35.91	32.55	7.35	0.77	3.14	20.28	2.71	0.47	8.92
MC500	5.45	76.25	12.03	2.36	0.42	2.82	6.12	2.35	0.38	117.75
MC600	5.75	77.03	13.57	1.29	0.34	2.37	5.40	1.14	0.30	103.42
MC700	6.20	78.76	14.02	1.12	0.27	2.18	3.65	0.96	0.20	106.00
MC800	6.77	78.81	15.37	0.77	0.16	2.24	2.66	0.60	0.13	98.79
MC900	7.03	79.98	15.50	0.44	0.10	2.39	1.60	0.34	0.08	105.81
Item	Al (%)	Fe (%)	Si (%)	Cl (%)	Ca (%)	Ti (%)	Na (%)	P (%)	Mg (%)	K (%)
MFS	20.56	7.44	3.98	1.95	0.83	0.65	0.26	0.08	0.10	0.05
MC500	43.89	16.94	9.04	2.07	1.85	1.40	0.55	0.19	0.22	0.11
MC600	44.16	17.37	9.29	1.70	1.92	1.50	0.56	0.19	0.23	0.12
MC700	45.89	17.57	9.33	1.29	2.06	1.53	0.55	0.19	0.24	0.11
MC800	45.96	17.65	9.46	0.97	2.07	1.60	0.58	0.19	0.23	0.10
MC900	47.13	17.95	9.56	0.57	1.96	1.63	0.62	0.18	0.25	0.10

#### 3.2.2 Mineral elements composition

**Fig. 4:**
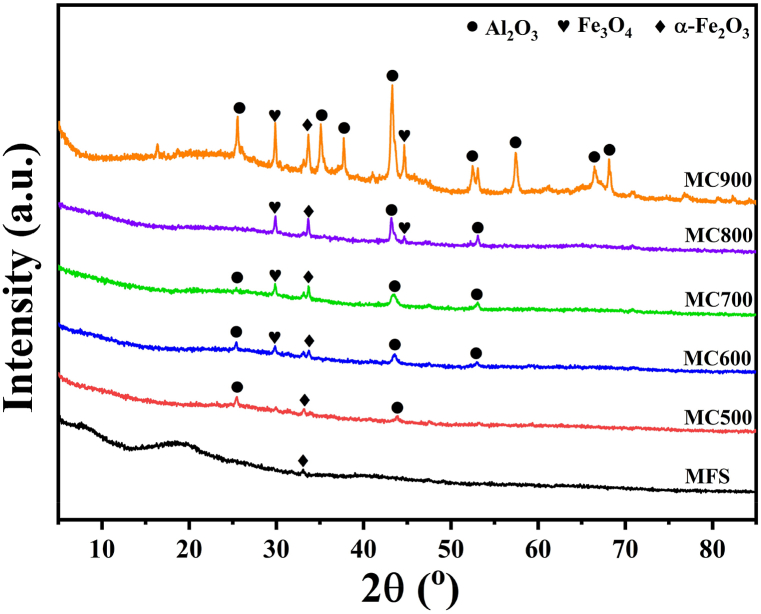
XRD patterns of MFS and its biochars.

The mineral elements composition in MFS and its biochars was analyzed using XRF, and the results are presented in Table 1. The raw MFS exhibited high levels of Al, along with several other abundant mineral elements, including Fe, Si, Cl, Ca, and Ti. It was observed that the Al and Fe contents increased from 20.56% to 7.44% in raw MFS to 43.89% and 16.94% in MC500 after MFS pyrolysis, and this increase was more pronounced with higher temperatures. Based on the mineral crystalline phases revealed in Fig. 4, it is evident that the biochars contained crystalline Al_2_O_3_, α-Fe_2_O_3_ and Fe_3_O_4_. Notably, the peaks associated with mineral crystalline became more distinct as the pyrolysis temperature increased. Therefore, Al and Fe were primarily exhibited in crystalline mineral forms and showed minimal release into the vapor phase. The high levels of Al and Fe oxides indicated the existence of acidic active sites in biochar. These active sites not only have the potential to promote dehydrogenation and hydrocracking reactions but also contribute to heavy metals immobilization during MFS pyrolysis.

#### 3.2.3 Surface morphology and pore structure

**Fig. 5:**
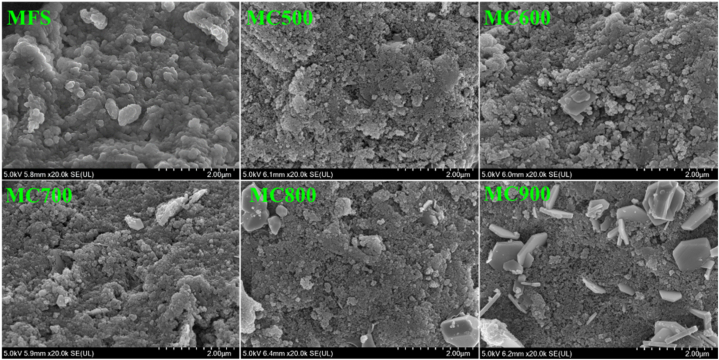
SEM images of MFS and its biochars.

SEM analysis was employed to assess the surface morphology of MFS and its biochars. As shown in Fig. 5, the surface of the MFS was relatively smooth with an abundance of small granules. In contrast to MFS, the surfaces of MC500 displayed a tendency to develop smaller, more regular structures and small granules. The grey granular crystals were initially found in MC600 and became increasingly pronounced with the pyrolysis temperature, particularly on the surface of MC900. Those crystals of minerals may function as a metal passivator to promote the enhancement of heavy metal immobilization through the creation of a matrix of crystal compounds [17]. As shown in Table 1, the BET surface area of MFS was only 8.92 m^2^/g. After MFS pyrolysis, the BET surface area of biochars significantly increased. Notably, MC500 exhibited the highest surface area at 117.75 m^2^/g, indicating a well-developed porous structure with relatively high specific surface areas. However, as the pyrolysis temperature rising from 500 to 900 °C, the BET surface area of the biochar underwent a slight decrease. This reduction can be attributed to the generation of tars during the thermal degradation of organic matter, which tends to adhere to the surface and impede the porous structure of the biochar. Furthermore, the formation of numerous mineral crystals may have played a role in reducing the surface area of biochar.

#### 3.2.4 Surface functional groups

**Fig. 6:**
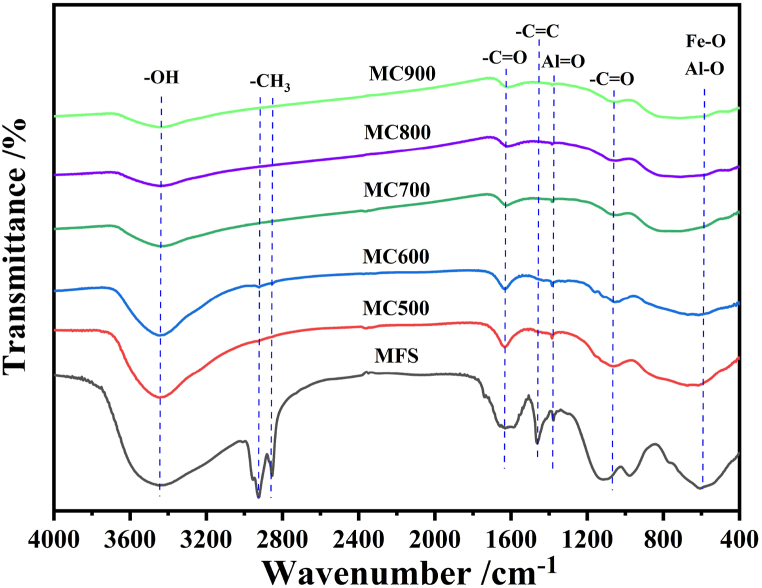
FTIR spectra of MFS and its biochars.

Fig. 6 presented the functional groups changes on the surface of MFS and biochars as examined via FTIR spectral analysis. The diminishing intensity of the –OH stretching vibration at 3400 cm^−1^ from MFS to biochars can be attributed to the decomposition of a significant number of hydroxyl groups during MFS pyrolysis. The peaks associated with –CH_3_ stretching vibration (occurring around 2800∼3000 cm^−1^) disappeared after the conversion of MFS into biochars, indicating the complete decomposition of methyl-containing organics. However, the intensity of C=C, C=O, and N–O stretching vibrations exhibited a slight decrease as the temperature increased, indicating the strong aromatic structure of biochars. The peaks around 1052 cm^−1^ and 450∼800 cm^−1^ corresponded to Fe–O and Al–O, respectively. Remarkably, these peaks remained relatively stable even with the increase in pyrolysis temperature, indicating a substantial accumulation of metal oxides in minerals present in the biochar. These findings were consistent with the results of XRD and SEM. The accumulation of mineral oxides in biochar have the potential to interact with a larger quantity of heavy metals in MFS, thereby enhancing its capacity to immobilize heavy metals.

### 3.3 Heavy metals analysis

#### 3.3.1 Total contents of heavy metals

Table 2 presented the contents of Zn, Cr, Pb, Ni, Cu, and Cd in MFS and its biochar. Notably, the heavy metals contents in TDS were significantly lower than those observed in other types of sludge [29,30]. The heavy metals concentrations in MFS follow a descending order: Zn > Cr > Pb > Ni > Cu > Cd. Among those metals in MFS, Zn exhibited the highest content, reaching a maximum of 147.55 mg/kg, possibly due to the widespread use of galvanized pipes in wastewater plants in China [18]. After MFS pyrolysis, the biochars showed varying degrees of heavy metal enrichment, primarily influenced by the specific heavy metal species and the pyrolysis conditions. For instance, the total contents of Zn, Cr, Ni, and Cu in biochar increased with rising pyrolysis temperatures from 500 to 700 °C. This increase was primarily attributed to the thermal decomposition of organic components and the accumulation of low-volatile heavy metals. However, these metals contents began to decrease as the pyrolysis temperature further increased from 700 to 900 °C, possibly influenced by the increasing significance of inherent minerals at higher temperatures. In contrast, the contents of Pb and Cd consistently decreased with rising pyrolysis temperatures due to their strong volatility.

**Table 2:** Total contents of heavy metals in MFS and its biochars.

Samples	Total contents (mg/kg)					
	Zn	Cr	Pb	Ni	Cu	Cd
MFS	147.55 ± 4.81	108.66 ± 3.28	28.38 ± 0.94	9.54 ± 0.48	9.77 ± 0.40	0.20 ± 0.01
MC500	319.35 ± 2.01	239.49 ± 5.65	59.15 ± 1.76	20.92 ± 0.44	19.82 ± 0.46	0.38 ± 0.04
MC600	295.37 ± 3.97	252.41 ± 1.72	58.55 ± 1.10	20.73 ± 0.41	20.06 ± 0.69	0.33 ± 0.02
MC700	297.07 ± 5.63	248.38 ± 1.25	52.73 ± 0.43	21.46 ± 0.18	20.95 ± 0.08	0.21 ± 0.00
MC800	196.99 ± 1.35	255.43 ± 3.80	42.12 ± 0.55	20.81 ± 0.47	20.83 ± 0.06	0.12 ± 0.01
MC900	107.53 ± 2.21	247.41 ± 4.13	22.92 ± 0.57	20.65 ± 0.31	19.78 ± 0.66	0.01 ± 0.00
Threshold values						
China pH < 6.5	2000	600	300	100	800	5
China pH ≥ 6.5	4000	1000	1000	200	1500	20

**Fig. 7:**
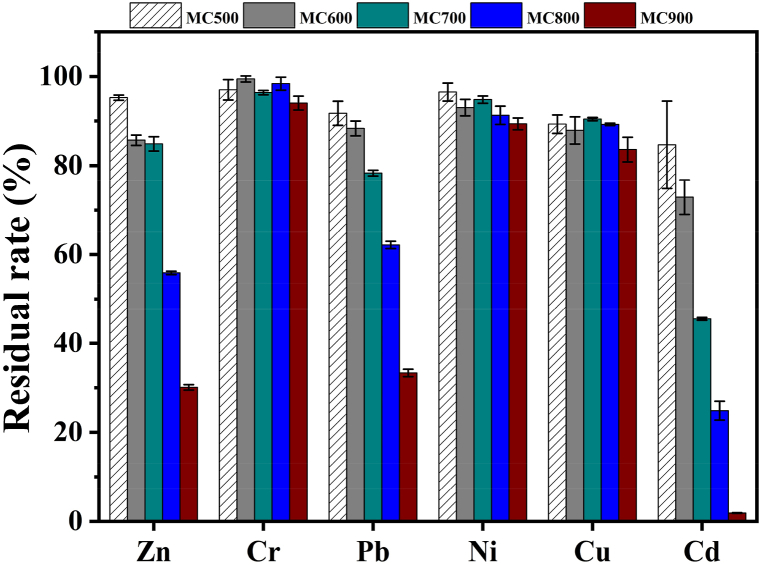
The residual rate of heavy metals in biochars.

Fig. 7 showed that the retention rates of Cr, Ni, and Cu in biochar exceeded 83.59%, primarily due to their low vapor pressures and high boiling points. In contrast, Zn, Pb, and Cd retention rates in biochars gradually decreased with rising temperature. For instance, the retention rates of Zn, Pb, and Cd in MC900 were only 30.09%, 33.35%, and 1.87%. The main reason for the lower retention of Zn, Pb, and Cd in biochars at higher temperatures is their strong volatility and the high levels of Cl content in raw MFS. This leads to the production of Cl_2_ and HCl, which can react with Zn, Pb, and Cd in MFS, forming easily volatilized metal chlorides [37]. Overall, despite an increase in the contents of some heavy metals in biochars, the total heavy metals contents in MFS and its biochars remained below the national standards when compared to the threshold values established for the use of sludge in forestland.

#### 3.3.2 TCLP leaching properties of heavy metals

**Fig. 8:**
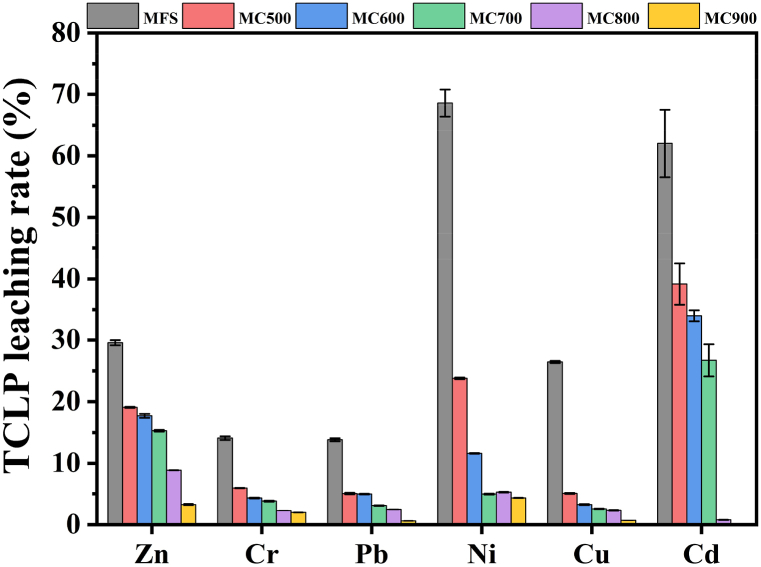
TCLP leaching rate of heavy metals in MFS and its biochars.

The TCLP leaching method simulates natural leaching conditions in a landfill to assess the direct toxicity of heavy metals in hazardous waste. As indicated in Table 3, the leaching contents of Zn, Cr, Pb, Ni, Cu, and Cd in raw MFS were 43.61, 15.31, 3.93, 6.54, 2.58, and 0.12 mg/kg, respectively. It is noteworthy that the leaching levels of Zn, Cr, and Ni in MFS exceeded the USEPA threshold values, suggesting that the direct disposal of MFS in landfills poses a significant environmental risk to both groundwater and soil, making it eligible for classification as hazardous waste under USEPA regulations. The pyrolysis process effectively decreased the leaching rate of heavy metals in biochars, and this reduction was observed to increase progressively with higher pyrolysis temperatures, as illustrated in Fig. 8. Correspondingly, the leaching contents of heavy metals gradually decreased with increasing temperature, except for Zn in MC500, NC600, and MC700, which exhibited a slight increase. It is worth emphasizing that the amounts of the six heavy metals leached from MC900 were found to be below the USEPA permissible limits, providing a clear demonstration of the reduced toxicity of heavy metal leaching in the biochar following the MFS pyrolysis process.

**Table 3:** TCLP leaching contents of heavy metals in MFS and its biochars.

Samples	TCLP contents (mg/kg)					
	Zn	Cr	Pb	Ni	Cu	Cd
MFS	43.61 ± 0.62	15.31 ± 0.33	3.93 ± 0.07	6.54 ± 0.21	2.58 ± 0.02	0.12 ± 0.01
MC500	60.95 ± 0.26	14.19 ± 0.04	2.98 ± 0.07	4.98 ± 0.03	1.00 ± 0.01	0.15 ± 0.02
MC600	52.29 ± 0.96	10.89 ± 0.19	2.90 ± 0.03	2.41 ± 0.01	0.65 ± 0.02	0.11 ± 0.02
MC700	45.43 ± 0.38	9.48 ± 0.20	1.62 ± 0.02	1.06 ± 0.02	0.53 ± 0.01	0.06 ± 0.01
MC800	17.40 ± 0.13	5.81 ± 0.02	1.04 ± 0.02	1.09 ± 0.02	0.48 ± 0.02	ND
MC900	3.49 ± 0.16	4.93 ± 0.13	0.14 ± 0.01	0.89 ± 0.02	0.14 ± 0.00	ND
Threshold values^a^	5	5	5	5	5	1

a From (EPA US, 1990).

## 4 Conclusions

High-quality pyrolytic products with low toxicity of heavy metals were prepared through pyrolysis of MFS under various temperatures. The effects of temperature and inherent minerals on the composition of pyrolysis products were studied. Increasing the pyrolysis temperature led to a decrease in biochar and oil yields, while remarkably increasing the gas yield with a maximum of 18.86 wt% at 900 °C. Inherent minerals at higher temperatures enhanced the oil and gas qualities through the facilitation of aromatic hydrocarbon formation, reduction of oxygenated and nitrogenated compounds, and increased yields of CO, CH_4_, and H_2_. Furthermore, it was worth noting that the inherent minerals have the potential to improve carbon sequestration and construct well-developed porous structures of carbonaceous materials. The MFS pyrolysis process also led to a reduction in the leaching contents of heavy metals in the biochar. Overall, the findings indicate that the pyrolysis process has the potential to not only improve the quality of pyrolysis products but also mitigate the toxicity of heavy metals in MFS.

## Funding statement

This work was supported by the 10.13039/501100015310Natural Science Foundation of Xinjiang Uygur Autonomous Region (No. 2022D01C737), the 10.13039/501100001809National Nature Science Foundation of China (No. 52000011 and 52260008), and the Natural Science Foundation of Changji University (No.22ky003).

## Data availability statement

Data will be made available on request.

## Additional information

No additional information is available for this paper.

## CRediT authorship contribution statement

**Guidan Zhu:** Writing – review & editing, Writing – original draft, Visualization, Validation, Resources, Project administration, Investigation, Funding acquisition, Formal analysis, Data curation. **Xingdong Wang:** Writing – review & editing, Validation, Supervision, Investigation, Data curation, Formal analysis. **Xuan Yin:** Methodology, Validation. **Mengmeng Zhu:** Resources, Validation. **Jiaying Li:** Investigation, Formal analysis. **Ling Cao:** Resources. **Zhiyang Sun:** Investigation. **Hehua Zeng:** Writing – review & editing, Supervision, Project administration, Funding acquisition, Validation.

## Declaration of competing interest

The authors declare the following financial interests/personal relationships which may be considered as potential competing interests: Guidan Zhu reports financial support was provided by 10.13039/501100015310Natural Science Foundation of Xinjiang Uygur Autonomous Region. Hehua Zeng reports financial support was provided by 10.13039/501100001809National Natural Science Foundation of China. Guidan Zhu reports financial support was provided by Natural Science Foundation of Changji University. If there are other authors, they declare that they have no known competing financial interests or personal relationships that could have appeared to influence the work reported in this paper.
